# DEMENTIA RISK REDUCTION SUMMIT: ADDRESSING DEMENTIA RISK ACROSS THE PREVENTION SPECTRUM

**DOI:** 10.1093/geroni/igae098.2270

**Published:** 2024-12-31

**Authors:** Chelsea Kline, Matthew Baumgart, Shelby Roberts

**Affiliations:** Alzheimer’s Association, Chicago, Illinois, United States; Alzheimer’s Association, Chicago, Illinois, United States; Alzheimer’s Association, Chicago, Illinois, United States

## Abstract

As the science around dementia risk reduction has evolved in the last 20 years, public health action to address the risk factors has become an increasing imperative. In May 2023, the Alzheimer’s Association Public Health Center of Excellence on Dementia Risk Reduction, funded by the CDC, held the first Dementia Risk Reduction Summit (Summit) at the CDC in Atlanta. The event convened 230 public health professionals, academics, and researchers with 43% of attendees representing state, local, and tribal health departments. The Summit included six sessions focusing on each level of the spectrum of prevention– (1) strengthening individual knowledge and skills, (2) promoting community education, (3) educating providers, (4) fostering coalitions and networks, (5) changing organizational practices, and (6) influencing policy and legislation – and showcased how public health could address risk factors for cognitive decline and dementia at each level. An additional session focused on social determinants of health. Initial evaluations of the Summit found most attendees would recommend the event to a colleague. Additionally, nearly all had taken action to address dementia risk within two months following the Summit (46%) or said they planned to (44%). 90% of attendees reported the Summit provided helpful information that could be applied to their organization’s approach to dementia risk reduction. Recordings of the Summit have been viewed 1,725 times. Additional follow-up surveys were sent to key health department officials in March 2024 to understand the long-term impact of the Summit. This structured national convening increased knowledge and confidence around dementia risk reduction.

